# Chronic disease risk factors among hospital employees: A cross-sectional study in Türkiye

**DOI:** 10.1371/journal.pone.0302910

**Published:** 2025-01-09

**Authors:** Volkan Medeni, Vildan Topcu, Fatma Bozdağ, İrem Medeni

**Affiliations:** 1 Department of Public Health, Faculty of Medicine, Gazi University, Ankara, Türkiye; 2 Employee Health Department, General Directorate of Public Health, Ministry of Health, Ankara, Türkiye; Universitas 17 Agustus 1945 - Jakarta, INDONESIA

## Abstract

**Introduction:**

Chronic diseases have become a significant public health problem with the prolongation of human life. There are four main behavioral risk factors for mortality. This study evaluated the significant risk factors for chronic diseases in university hospital employees.

**Materials and methods:**

The cross-sectional study population consisted of hospital employees working at Gazi University Hospital for at least one year. The sample size was calculated to be 285, with a 100% response rate. The study’s independent variables were age, gender, educational status, working department, and presence of chronic diseases. Dependent variables were smoking, alcohol use, physical inactivity, and body mass index (BMI) categories. Data on participants’ characteristics, habits, and behaviors were obtained from the hospital system or with open-ended questions. Their body weight and height were measured. The International Physical Activity Questionnaire (IPAQ) was used to assess physical inactivity.

**Results:**

The smoking prevalence of hospital employees was 41.8%. Regular alcohol use was 19.3%. Based on the BMI values obtained, 37.9% of the participants were pre-obese, and 18.2% were obese. According to the results of the IPAQ, 13.7% were inactive. The prevalence of smoking was 50.4%, alcohol consume 11.6%, physical inactivity 50.4%, and overweight 65.3% among those who graduated from high school or lower. In contrast, the prevalences were 35.4%, 25.0%, 69.5%, and 49.4%, respectively, among those who graduated from university or higher. A one-unit increase in age of participants without chronic disease increased BMI by 1.06 times (p<0.05). When individuals with a high school education or lower are taken as the reference group, it was found that physical inactivity is 1.78 times higher among those with a university degree or higher (p<0.05).

**Conclusion:**

The effect of education level on health habits and behaviors should be considered in terms of the target group and content of preventive health programs and awareness-raising studies.

## Introduction

Chronic diseases are non-communicable diseases that occur as a result of a combination of genetic, physiological, environmental, and behavioral factors and have become a significant public health problem with the prolongation of human life. These diseases, which cause about three-quarters of the deaths in the world, include cancers, cardiovascular diseases, diabetes, and chronic respiratory diseases [1]. Smoking, alcohol consumption, physical inactivity, and unhealthy diet are the four primary behavioral risk factors for chronic disease mortality [2].

World Health Organisation (WHO) recognizes that the risk of chronic disease can be reduced by making changes to four behaviors (tobacco use, alcohol use, unhealthy diet, and physical inactivity) and by changing metabolic risk factors such as high blood pressure or cholesterol [3]. Management of chronic diseases is a priority of the public health sector in most countries. At the global level, WHO and United Nations agencies are working together to design policies and strategies for the risk of chronic diseases [4]. Nowadays, chronic diseases have a significant negative impact on the health and economies of developed and developing countries [5]. It is known that one-quarter of the working-age population in Europe has a chronic disease. Premature death from chronic diseases in the working-age population causes economic losses of 115 billion Euros every year in Europe [6].

Currently, about half of the United States population has a chronic disease, and 86% of healthcare costs are due to chronic diseases [7]. Approximately 90% of deaths and 85% of years lived with disability in the WHO European Region are due to chronic disease [8]. In Turkey, death due to chronic diseases has a frequency of 90%, and possibly premature death due to chronic diseases has a frequency of 16%. Chronic diseases cause significant problems for individuals, families, society, and the health system. Prevention and control of risk factors become essential due to the economic burden they create [9].

Physical inactivity, one of the risk factors of chronic diseases, refers to the lack of sufficient physical activity, including all kinds of movements that cause energy expenditure. Physical activity includes exercise, occupation, leisure time, and housework [10]. It is effective in preventing chronic diseases and related complications. Moderate and above physical activity is reported to be associated with lower mortality and morbidity. In recent years, the frequency of physical inactivity in society has gradually increased [11]. Physical inactivity is also closely associated with overweight.

Obesity is a multifactorial disease defined as abnormal or excessive fat accumulation that poses a health risk. Genetics, unhealthy eating habits, and sedentary lifestyles are risk factors for obesity. It has been determined that decreased physical activity is more effective than increased energy intake in developing obesity [12]. Anthropometric methods and calculation of body mass index assess excess adiposity. Body mass index is calculated by dividing body weight in kilograms by square height in meters. People with a body mass index of 25 and above are defined as overweight, and those with a body mass index of 30 and above as obese [13]. Overweight and obesity cause many chronic diseases such as diabetes, coronary artery disease, hypertension, stroke, cancer, and psychological problems such as depression, stress, and anxiety, and negatively affect the quality of life of the person [14].

Alcohol use and smoking are among the risk behaviors for the development of chronic diseases. Alcohol use is associated with diabetes, coronary artery disease, and many types of cancer. Cancers and chronic gastrointestinal diseases are common causes of alcohol-related death and disability [15]. Smoking is a chronic disease risk factor that is highly addictive among smokers and is particularly associated with chronic respiratory diseases. Long-term smokers die ten years earlier on average. The risk of coronary disease, chronic obstructive pulmonary disease, lung cancer, and stroke is increased in smokers compared to non-smokers [16].

It is estimated that chronic diseases are frequently seen in healthcare workers as well as in society. Healthcare workers experience significant stress due to excessive workload, challenges in healthcare delivery, mismatches between services and personnel, and workplace-specific risks. The intense and demanding work environment, along with poor sleep quality and limited physical activity, contributes to numerous health issues, particularly obesity. Additionally, long shifts disrupt circadian rhythms, increasing the risk of chronic diseases. Although healthcare workers are assumed to be more aware of the risks of obesity, studies indicate they are at a higher risk of being overweight or obese compared to the general population [17–20]. Despite these risk factors, studies evaluating more than one chronic disease risk factor in hospital workers are almost non-existent. Widespread research on this topic could help these workers become aware of the factors that threaten their health. By identifying and addressing modifiable risk factors such as excessive weight, physical inactivity, smoking, and alcohol use, the emergence of chronic diseases and complications can be prevented. This way, hospital employees can improve their job performance and support their colleagues and patients in adopting a healthier lifestyle. This study aimed to evaluate the main chronic disease risk factors in healthcare workers in a university hospital.

## Methods

Our cross-sectional study was conducted among non-physician employees at Gazi University Hospital in 2023. The population of the study consisted of 3304 hospital employees except physicians. Physicians in this hospital were a few academicians and had very different risk factors from hospital employees. Therefore, they were excluded from the study. Hospital employees who had worked at Gazi University Hospital for at least one year and volunteered to participate in the study were included. The sample size was determined according to the smoking prevalence of 28.3% in the Turkish Health Survey [21].

The sample size to be selected is calculated as follows:

N (Number of people in the universe) = 3304

n = [Nt^2^pq] / [d^2^ (N-1) + t^2^ pq]

= [3304 x (1.96) ^2^ x 0.283 x 0.717] / [0.05^2^ x (3304–1) + (1.96) ^2^ x 0.283 x 0.717] = 285 people

t (value found for the largest degree of freedom in the t-table at a 95% confidence level) = 1.96

p (smoking prevalence) = 0.283

q (probability that the event under study will not occur) (1-p) = 0.717

d (error) = 0.05

After the employees were weighted according to gender and age, the sample to be taken from each stratum was determined. The employee list was sorted according to gender and age, and the participants were determined by systematic sampling. When 285 people were aimed to be reached, the inter-personal interval was 3304/285 = 11.59. A lottery was drawn to determine the starting point, and the following persons were reached by adding 12 to the determined number. People who did not accept to participate in the study or did not meet the inclusion criteria were replaced by other people of similar age and gender. This way, the study was conducted with 285 people, and the response rate was 100%. People who refused to participate in the study, physically disabled people, pregnants, who had an operation in the last month, and who were mentally unable to complete the questionnaire were excluded from the study. Informed consent forms were obtained from the participants. The study received ethical approval at the Gazi University Ethics Commission meeting dated 21.11.2023 and numbered 20; the research code is 2023–1412.

The study’s data collection form consists of three sections. In the first part, sociodemographic characteristics were analyzed. In the second part, chronic diseases and risk factors were questioned. The third part included a short form of the International Physical Activity Questionnaire (IPAQ). The study’s independent variables were age, gender, educational status, working department, and presence of chronic diseases. Dependent variables were smoking, alcohol use, physical inactivity, and BMI. Collecting data took 8–10 minutes per participant.

The age, gender, graduation status, and occupation data of the participants were obtained from the computer system. Information about place of work, presence of chronic diseases, smoking, and alcohol use were obtained by open-ended questions. Body weight measurements to determine body-mass indexes were performed by removing heavy clothes, and height measurements were performed without shoes. The IPAQ used to assess physical inactivity was developed by Craig et al. [22]. Öztürk performed the Turkish validity and reliability study of the short form in 2005 [23]. The form provides information about the time spent walking, sitting, moderate intensity, and vigorous physical activities. The duration and frequency of walking, moderate intensity, and vigorous physical activities are used in scoring. According to the results, people are classified as inactive, minimally active, and very active.

During statistical analysis, participants were grouped as under 40 and over 40; those with university, master’s, and doctorate degrees were grouped as university and above, and others were grouped as high school and below. Nurses, maintenance workers, medical secretaries, health technicians, etc., are considered "health workers"; cleaners, technical personnel, security, administrative personnel, etc., are categorized as "other personnel." "Other personnel" refers to professionals who play a specific role in healthcare services but are not classified as healthcare professionals. Those who do not smoke and have quit smoking are considered non-smokers, and those who do not drink alcohol and have quit drinking alcohol are considered not alcohol consumer.

BMI categories are underweight: <18.5 kg/m^2^, normal: 18.5–24.9 kg/m^2^, pre-obese: 25–29.9 kg/m^2^, obese >30. In our study, those with BMI≥25 kg/m^2^ were expressed as overweight.

The calculation of the total score of the IPAQ short form includes the sum of the duration and frequency of walking, moderate-intensity activity and vigorous activity. The energy required for activities is calculated with the MET-minute score.

Weekly physical activity levels are calculated using these values:

Walking MET-min/week = 3.3 x walking minutes x days per week

Moderate intensity MET-min/week = 4.0 x moderate intensity activity minutes x days per week

Vigorous MET-min/week = 8.0 x vigorous activity minutes x days per week

Total, MET-min/week = (walking + moderate intensity + vigorous) MET-min/week

Accordingly, there are three activity categories:

1. Low: Situations that cannot be included in categories 2 and 3.
2. Moderate: a) 3 or more days of at least 20 minutes of vigorous activity
      b) 5 or more days of moderate-intensity activity or walking for at least 30 minutes per day
      c) 5 or more days of a combination of walking and moderate-intensity activity that provides a minimum of 600 MET-min/week
3. High: a) At least 3 days of vigorous activity or more days that provide a minimum of 1500 MET-min/week
      b) 7 or more days of walking, moderate-intensity, or vigorous activity that provides a minimum of 3000 MET-min/week.

In our study, those in moderate and high categories were considered physically active.

The statistical analyses were performed using Statistical Package for Social Science for Windows 23.0. In descriptive findings, categorical variables were expressed as numbers and percentages; continuous variables were expressed as mean, standard deviation, and minimum-maximum values. Categorical variables were analyzed using Pearson and Fisher’s exact chi-square tests. Binary logistic regression analysis was performed with the significant variables in single analyses. Statistical significance was accepted as p<0.05.

## Results

The study included 285 participants, with a distribution across various age groups: 22.1% were under 30 years old, 27.7% were between 30–39, 34.8% were between 40–49, and 15.4% were aged 50–59. In terms of gender, 59.6% were female, and 40.4% were male. Regarding education levels, 4.2% had completed elementary school, 6.3% had finished middle school, 32.0% were high school graduates, 48.4% had a college degree, and 9.1% held a Master’s or PhD. Occupationally, the largest groups were nurses (30.9%), followed by cleaning workers (16.9%) and caregivers (14.3%). Other roles included medical secretaries (13.3%), health technicians (9.5%), and various other health-related or support positions. Participants worked in various hospital departments, with the highest representation from clinics (26.3%), outpatient clinics (16.1%), and intensive care units (13.0%), among other departments (Table 1).

**Table 1:** Sociodemographic characteristics of the participants, Türkiye, 2023.

	Chronic Disease (-)	Chronic Disease (+)	Total
	n (%)	n (%)	n (%)
**Age group (n = 285)**			
Less than 30 years old	55 (27.0)	8 (9.9)	63 (22.1)
30–39 years old	60 (29.4)	19 (23.5)	79 (27.7)
40–49 years old	66 (32.4)	33 (40.7)	99 (34.8)
50–59 years old	23 (11.2)	21 (25.9)	44 (15.4)
**Gender (n = 285)**			
Female	119 (58.3)	51 (63.0)	170 (59.6)
Male	85 (41.7)	30 (37.0)	115 (40.4)
**Graduation status (n = 285)**			
Elementary school	7 (3.4)	5 (6.2)	12 (4.2)
Middle school	10 (4.9)	8 (9.9)	18 (6.3)
High school	70 (34.3)	21 (25.9)	108 (32.0)
College	100 (49.0)	38 (46.9)	138 (48.4)
Master’s degree/PhD	17 (8.3)	9 (11.1)	26 (9.1)
**Occupation (n = 285)**			
Nurse	62 (30.4)	26 (32.1)	88 (30.9)
Caregiver	25 (12.3)	16 (19.8)	41 (14.3)
Medical Secretary	23 (11.3)	15 (18.5)	38 (13.3)
Health technician	21 (10.3)	6 (7.4)	27 (9.5)
Other health personnel *	12 (5.9)	2 (2.5)	14 (4.9)
Cleaning worker	37 (18.1)	11 (13.6)	48 (16.9)
Technical staff	7 (3.4)	3 (3.7)	10 (3.5)
Security	9 (4.4)	1 (1.2)	10 (3.5)
Administrative staff	6 (2.9)	1 (1.2)	7 (2.5)
Other staff **	2 (1.0)	0 (0.0)	2 (0.7)
**Department (n = 285)**			
Clinic	56 (27.5)	19 (23.5)	75 (26.3)
Outpatient clinic	30 (14.7)	16 (19,8)	46 (16.1)
Intensive care	30 (14.7)	7 (8.6)	37 (13.0)
Administrative unit	17 (8.3)	11 (13.6)	28 (9.8)
Imaging/Laboratory	20 (9.8)	5 (6.2)	25 (8.8)
Operating room	15 (7.4)	9 (11.1)	24 (8.4)
Emergency service	13 (6.4)	3 (3.7)	16 (5.6)
Technical service	7 (3.4)	4 (4.9)	11 (3.9)
Security booth	8 (3.9)	1 (1.2)	9 (3.1)
Cafeteria	4 (2.0)	2 (2.5)	6 (2.1)
Other ***	4 (2.0)	4 (4.9)	8 (2.8)

* Pharmacist, dietician, physiotherapist, laborant, biologist, chemist, psychologist

**Driver, tailor

*** Pharmacy, medical equipment, morgue, transportation

Among the 285 participants, 81 were diagnosed with at least one chronic disease. Specifically, 23 participants had diabetes mellitus, 23 had hypertension, 25 had thyroid disorders, and 4 had hypercholesterolemia. Additionally, 15 participants suffered from other cardiovascular diseases, 9 from respiratory diseases, 13 from neurological disorders, 6 from rheumatologic conditions, 6 from musculoskeletal disorders, 4 from cancer, 3 from gastrointestinal diseases, and 4 experienced hearing loss.

Regarding health behaviors, 41.8% of employees were smokers, while 19.3% reported regular alcohol consumption, and 71.9% had never consumed alcohol. In terms of BMI, 37.9% of participants were categorized as pre-obese and 18.2% as obese. Based on the IPAQ results, 13.7% were physically inactive. No statistically significant associations were found between smoking, alcohol use, BMI, and physical activity levels with the presence of chronic diseases (Table 2).

**Table 2:** Chronic disease risk factors of the participants, Türkiye, 2023.

	Chronic Disease (-)	Chronic Disease (+)	p value*	Total
	n (%)	n (%)		n (%)
**Smoking (n = 285)**			0.345	
Never smoked	99 (48.5)	35 (43.2)		134 (47.0)
Quit smoking	25 (12.3)	7 (8.6)		32 (11.2)
Smokes	80 (39.2)	39 (48.2)		119 (41.8)
**Alcohol use (n = 285)**			0.286	
Never used alcohol	162 (79.4)	63 (77.8)		205 (71.9)
Stopped using alcohol	2 (1.0)	3 (3.7)		25 (8.8)
Regularly uses alcohol	40 (19.6)	15 (18.5)		55 (19.3)
**Body-Mass Index (n = 285)**			0.074	
Underweight	3 (1.5)	1 (1.2)		4 (1.4)
Normal	90 (44.1)	33 (40.7)		121 (42.5)
Pre-obese	82 (40.2)	25 (30.9)		108 (37.9)
Obese	29 (14.2)	22 (27.2)		52 (18.2)
**Physical inactivity (n = 285)**			0.518	
Inactive	25 (12.3)	14 (17.3)		39 (13.7)
Minimal active	98 (48.0)	38 (46.9)		136 (47.7)
Active	81 (39.7)	29 (35.8)		110 (38.6)

*: *Chı-Square Test*

Among participants under 40, 40.1% were smokers, 24.6% reported alcohol use, 59.9% were physically inactive, and 42.3% were overweight. For those aged 40 and above, these rates were 43.4%, 14.0%, 62.9%, and 69.9%, respectively. Smoking prevalence was 42.4% in women and 40.9% in men, while alcohol consumption was higher in men (25.2%) than in women (15.3%). Physical inactivity was similar between genders, at 61.2% for women and 61.7% for men, but overweight prevalence was higher in women (61.8%) compared to men (47.8%). Significant differences were noted in alcohol consumption and overweight status by age and gender, both overall and in participants with chronic diseases (p<0.05). Participants with a high school education or lower had smoking and alcohol use rates of 50.4% and 11.6%, respectively, with 50.4% being physically inactive and 65.3% overweight. In contrast, university graduates had lower smoking rates (35.4%), higher alcohol use (25.0%), greater physical inactivity (69.5%), and a lower overweight prevalence (49.4%). Educational level significantly influenced all chronic disease risk factors (p<0.05), with noteworthy differences in alcohol use, physical inactivity, and overweight among those with chronic diseases (Table 3).

**Table 3:** Associations between participants’ sociodemographic characteristics and chronic disease risk factors, Türkiye, 2023.

	Total				Chronic Disease (-)				Chronic Disease (+)			
	Smoking	Alcohol use	Physical inactivity	Over-weight	Smoking	Alcohol use	Physical inactivity	Over-weight	Smoking	Alcohol use	Physical inactivity	Over-Weight
	n (%)	n (%)	n (%)	n (%)	n (%)	n (%)	n (%)	n (%)	n (%)	n (%)	n (%)	n (%)
**Age (n = 285)**												
Less than 40 years (n = 142)	57 (40.1)	35 (24.6)	85 (59.9)	60 (42.3)	44 (38.3)	28 (24.3)	67 (58.3)	46 (40.0)	13 (48.1)	7 (25.9)	18 (66.7)	12 (44.4)
40 years and over (n = 143)	62 (43.4)	20 (14.0)	90 (62.9)	100 (69.9)	36 (40.4)	12 (13.5)	56 (63.9)	65 (73.0)	26 (48.1)	8 (14.8)	34 (63.0)	35 (64.8)
*p-value*	0,582	**0,023**	0,594	**<0,001**	0.751	0.053	0.500	**<0.001**	0.999	0.225	0.743	0.080
**Gender (n = 285)**												
Female (n = 170)	72 (42.4)	26 (15.3)	104 (61.2)	105 (61.8)	48 (40.3)	20 (16.8)	73 (61.3)	68 (57.1)	24 (47.1)	6 (11.8)	31 (60.8)	34 (66.7)
Male (n = 115)	47 (40.9)	29 (25.2)	71 (61.7)	55 (47.8)	32 (37.6)	20 (23.5)	50 (58.8)	43 (50.6)	15 (50.0)	9 (30.0)	21 (70.0)	13 (43.3)
*p value*	0,803	**0,037**	0,924	**0,020**	0.698	0.233	0.717	0.354	0.798	**0.041**	0.403	**0.040**
**Graduation status (n = 285)**												
High school and below (n = 121)	61 (50.4)	14 (11.6)	61(50.4)	79 (65.3)	41 (47.1)	12 (13.8)	46 (52.9)	52 (59.8)	20 (58.8)	2 (5.9)	15 (44.1)	25 (73.5)
University and above (n = 164)	58 (35.4)	41 (25.0)	114 (69.5)	81 (49.4)	39 (33.3)	28 (23.9)	77 (65.8)	59 (50.4)	19 (40.4)	13 (27.7)	37 (78.7)	22 (46.8)
*p-value*	**0,011**	**0,005**	**0,001**	**0,008**	**0.046**	0.071	0.062	0.185	0.102	**0.013**	**0.001**	**0.016**
**Occupation (n = 285)**												
Healthcare workers (n = 208)	82 (39.4)	44 (21.2)	132 (63.5)	95 (45.7)	51 (35.7)	30 (21.0)	87 (60.8)	76 (53.1)	31 (47.7)	14 (21.5)	45 (69.2)	36 (55.4)
Other personel (n = 77)	37 (48.1)	11 (14.3)	43 (55.8)	30 (39.0)	29 (47.5)	10 (16.4)	36 (59.0)	35 (57.4)	8 (50.0)	1 (6.3)	7 (43.8)	11 (68.6)
*p-value*	0,190	0,192	0,241	0,311	0.112	0.450	0.808	0.579	0.869	0.158	0.057	0.332

Logistic regression analysis was performed to assess factors influencing BMI, physical inactivity, alcohol use, and smoking as chronic disease risk factors. Each additional year of age was associated with a 1.06-fold increase in BMI (p<0.05). No significant associations were found between BMI and gender or education level (p>0.05). Participants with a college degree were 2.3 times more likely to be physically inactive and 2.4 times more likely to consume alcohol compared to those with a high school education or less (p<0.05). Additionally, high school graduates were 1.825 times more likely to smoke than participants with a university degree or higher (p<0.05). Age and gender showed no significant associations with physical inactivity, alcohol use, or smoking (p>0.05) (Table 4).

**Table 4:** Logistic regression analysis of participants’ chronic disease risk factors, Türkiye, 2023.

	Total					Chronic Disease (-)					Chronic Disease (+)				
	Beta	SE^3^	P	Exp (B)	95% CI^4^	Beta	SE^3^	p	Exp (B)	95% CI^4^	Beta	SE^3^	p	Exp (B)	95% CI^4^
**Body-Mass Index**															
***Age***	0.057	0.013	**<0.001**	1.058	1.031–1.086	0.055	0.016	**0.001**	1.057	1.024–1.090	0.084	0.030	**0.005**	1.087	1.026–1.152
***Gender***															
Female	0.349	0.255	0.171	1.418	0.860–2.337	0.129	0.299	0.666	1.138	0.634–2.043	0.934	0.533	0.080	2.544	0.896–7.226
Male (ref)				1.0											
***Graduation status***															
High school and below^1^	0.476	0.257	0.063	1.610	0.974–2.662	0.257	0.299	0.738	0.390	0.720–2.323	-0.950	0.539	0.078	0.387	0.134–1.112
University and above^2^ (ref)				1.0											
**Physical inactivity**															
***Age***	0.015	0.012	0.218	1.015	0.991–1.040	0.013	0.015	0.395	1.013	0.983–1.044	0.016	0.025	0.509	1.016	0.968–1.067
***Gender***															
Female				1.0		0.048	0.297	0.871	1.049	0.587–1.877	-0.035	0.539	0.949	0.966	0.336–2.777
Male	0.026	0.256	0.918	1.027	0.622–1.694				1.0					1.00	
***Graduation status***															
High school and below^1^ (ref)				1.0											
University and above^2^	0.852	0.253	**0.001**	2.344	1.429–3.845	0.577	0.295	0,050	1.780	0.999–3.172	1.586	0.520	**0.002**	4,884	1.762–13.533
**Alcohol use**															
***Age***	-0.020	0.016	0.216	0.980	0.950–1.012	-0.017	0.019	0.395	0.984	0.947–1.022	-0.038	0.033	0.247	0.962	0.902–1.027
***Gender***															
Female (ref)				1.0											
Male	0.572	0.309	0.064	1.771	0.967–3.244	0.405	0.362	0.263	1.500	0.737–3.051	0.954	0.622	0.125	2.595	0.767–8.777
***Graduation status***															
High school and below^1^ (ref)				1.0											
University and above^2^	0.886	0.340	**0.009**	2.424	1.244–4.723	0.656	0.384	0.088	1.927	0.908–4.090	1.590	0.818	0.052	4.901	0.986–24.373
**Smoking**															
***Age***	0.006	0.012	0.595	1.006	0.983–1.031	0.002	0.015	0.919	1.002	0.972–1.032	0.001	0.023	0.981	1.001	0.956–1.047
***Gender***															
Female	0.021	0.250	0.932	1.022	0.626–1.667	-0.132	0.Ş299	0.659	0.877	0.488–1.574	0.328	0.487	0.501	1.388	0.535–3.601
Male (ref)				1.0											
***Graduation status***															
High school and below^1^	0.602	0.247	**0.015**	1.825	1.126–2.959	0.579	0.295	**0.049**	1.784	1.001–3.179	0.824	0.480	0.086	2.279	0.889–5.844
University and above^2^ (ref)				1.0											

^**1**^Elementary school, middle school, high school

^2^College, Master’s degree/PhD

^3^ SE: Standard error

^4^CI: Confidence interval

## Discussion

In our cross-sectional study conducted in a university hospital, 285 hospital employees were reached, and chronic disease risk factors such as overweight, physical inactivity, smoking, and alcohol consumption were analyzed. Determining the risk factors of chronic diseases, which are common in hospital employees as well as in the general population and which can negatively affect the general health status of the employees, will shed light on the studies aimed at eliminating these factors. As a result, it is essential in terms of preventing chronic diseases.

According to our findings, 41.8% of the participants were smokers. In a study conducted on healthcare workers in Spain, 40.0% of the participants smoked [24]. In a study conducted in Saudi Arabia, the prevalence of smoking in healthcare workers was 18.4% [25]. In a study conducted in Indonesia, the prevalence of smoking among healthcare workers was approximately one-fifth [26]. The prevalence of current smoking among working adults in the United States of America was 22.8% in men and 18.3% in women [27]. In another study conducted in Turkey, %39 of non-physician healthcare workers either smoke at the moment or have smoked in the past [28]. In the adult population in Turkey, 44.8% of men and 18.1% of women use tobacco products [29]. In Turkey, male healthcare workers smoke as much as the national average, female healthcare workers smoke more than the national average, and all healthcare workers smoke more than healthcare workers in other countries [30]. In our study, a higher result was found than in the literature. These results may suggest that healthcare workers in countries where smoking prevalence is high are exposed to more strenuous work tempo and stressful working conditions and use smoking as a tool to cope with these problems. In some countries, laws restricting smoking, inspections, and anti-smoking campaigns may explain the frequency differences. Cultural, economic, and social differences at the local level between countries should also be considered when interpreting study results.

In our study, 56.1% of the participants were pre-obese or obese. In a study on healthcare workers in the United Kingdom, 63,1% of participants were pre-obese or obese [31]. In another study conducted in China, 34.3% of the participants were pre-obese, and 11.2% were obese [32]. In South Africa, approximately 75% of health workers are pre-obese or obese [33]. In another study conducted in Turkey, almost half of the healthcare workers were pre-obese or obese [34]. In our study, the results were found to be high, similar to the literature. The prevalence of overweight and obesity in hospital workers may be explained by the fact that working long hours at a desk, especially in certain occupational groups, leads to a sedentary lifestyle. The high prevalence of pre-obesity and obesity in hospital staff who are not physically inactive may be due to reasons such as adverse effects on metabolism and eating habits due to intense and stressful working conditions, eating unhealthy snacks during night work, and sleep irregularity. In addition, differences in age, gender, income status, sociocultural characteristics, and behavioral factors between the samples of the mentioned studies may also play a role in the development of differences between the prevalence of pre-obesity and obesity.

In our study, 61.4% of the participants were not physically active. In a study conducted in Greece, 83.6% of healthcare workers were found to be low or moderately physically active [35]. In a study conducted in Spain on primary health care personnel, approximately 75% reported low levels of physical activity [36]. In a study conducted in Hungary, 77.2% of participants were not physically active [37]. In another study conducted in Turkey, 74.6% of the participants were not physically active [38]. The level of physical inactivity in our study is lower than the findings in the literature. It may be because physicians were not included in our study. These results may be explained by the fact that allied health personnel are more active than physicians. Depending on their job roles, they may exert more physical effort throughout the day, use more energy, and maintain higher levels of physical activity.

In this study, no significant differences were found between individuals with and without chronic disease in terms of smoking, alcohol consumption, BMI, and physical inactivity. This may be because individuals with chronic disease often reduce risky behaviors due to health concerns, minimizing group differences. In addition; genetic, environmental, or socioeconomic factors may overshadow these behaviors’ impact on chronic disease development. This finding suggests that chronic disease may involve complex factors beyond individual risk behaviors. Similar rates of risky behaviors in both groups could also reflect a generally low level of healthy lifestyle habits in the population. Our results emphasize the need to strengthen preventive health services for healthcare workers and raise awareness of risk factors.

In this study, 69.9% of participants aged 40 years and over and 42.3% of participants aged under 40 years were pre-obese or obese. The body mass index increased 1.058 times with a one-unit increase in the age of the participants. In a study among healthcare workers from Kenya, the prevalence of pre-obesity and obesity was 54.5% in the 30–39 age group, 80.0% in the 40–49 age group, and 88.2% in the 50–59 age group [39]. In a Nigerian study at a tertiary healthcare institution, individuals under the age of 44 were less likely to be associated with obesity [40]. In the United States, more than 30 percent of the population aged 60 years and older is obese [41]. In another study in Turkey, obesity started to increase in the 35–44 age group and reached the highest rate in the 45–54 age group [42]. Our results are consistent with the literature. In recent years, the prevalence of obesity in the elderly has reached an essential level in healthcare workers as well as in society. Complex interactions of many factors, such as decreased basal metabolic rate and physical activity with aging, decreased energy expenditure, sedentary lifestyle, and dietary changes, may lead to overweight.

In our study, 35.4% of individuals with university or higher education smoke, and 25.0% consume alcohol. Among those with a high school education or lower, 50.4% smoke, and 11.6% consume alcohol. A study conducted in Denmark found that those with the lowest level of education were frequent smokers, heavy drinkers, obese, and physically inactive [43]. A study conducted in the United States observed that lower levels of education were associated with more pack years of smoking and fewer attempts to quit [44]. A study conducted in England found that people in higher-income groups consumed alcohol more frequently. Although low-income individuals consumed alcohol less frequently, they consumed excessive amounts of alcohol when they did [45]. According to the WHO, there is a significant relationship between alcohol use and income level. Widespread and risky consumption is higher in the upper-income group worldwide [46]. Our findings are consistent with other studies in the literature. Since individuals with lower levels of education generally have lower incomes and because of the cost of alcohol, it is natural that more educated individuals use more alcohol. However, alcohol dependence and excessive use are also more common in people with lower levels of education worldwide. The situation is different for smoking. Individuals with lower levels of education may be prone to smoking due to reasons such as family, friends, physical environment, and sociocultural and psychological factors. Hospital workers generally include people with higher levels of education. However, low-income occupational groups such as cleaners, security guards, technical staff, drivers, and tailors also work in hospitals. In this respect, our findings may guide vocational training programs, support services, and promoting healthy living within the scope of combating smoking and alcohol in hospitals.

In our study, 50.4% of hospital workers with a high school education or below and 69.5% of healthcare workers with a university education or above were physically inactive. A study conducted on healthcare workers in Brazil observed that physical activity in leisure time decreased as the level of education decreased [47]. In another study conducted on healthcare workers in Turkey, when the relationship between education and physical activity level was analysed, it was found that physical activity level increased as education decreased [48]. The different results between the literature and our study are related to the inclusion or exclusion of activities in work life when asking about physical activity. The reason for the decrease in physical activity as the level of education decreases in Brazil is that only leisure time activities are questioned. Individuals with lower education levels among hospital workers are engaged in more strenuous and labor-demanding jobs during the day, work without having time to sit and rest during working hours, and perform more physical activity.

In our study, 65.3% of individuals with lower levels of education and 49.4% of those with university and above were found to be overweight. In a study conducted in Saudi Arabia, the risk of obesity was higher in illiterate and primary school graduates than in participants with university and higher education [49]. In a study conducted in Korea, the prevalence of obesity was higher in those with lower levels of education and household income [50]. Our results are consistent with the literature. It refutes the claims that as the level of education decreases, income decreases, and the difficulty of accessing food increases, obesity will decrease. The fact that individuals with lower levels of education are less conscious about healthy and balanced nutrition or eat a carbohydrate-dominant and protein-poor diet due to their low income can lead to unhealthy weight gain, and obesity may be observed more frequently in these individuals.

Our study has some limitations. It covers only healthcare professionals working in a hospital, and the findings could be more generalizable if conducted as a multicenter study across various hospital settings. Additionally, self-reported data on behaviors such as smoking and alcohol use might be influenced by social desirability bias, as some workers may have underreported due to stigma, religious beliefs, or fear of judgment. To mitigate this, confidentiality was assured, and the surveys were administered with an emphasis on anonymity to encourage honest responses. However, the potential impact of these biases remains a limitation. In the first stage, 48 of the 285 selected participants did not participate for various reasons. To address this, we included substitute participants with similar characteristics, which helped maintain the study sample’s representativeness.

## Conclusion

In our study, more than a quarter of healthcare workers have chronic diseases, 41% of the participants smoke, and 19.3% consume alcohol. Additionally, more than half of the participants had BMI ≥ 25. Various statistical differences were found between age, gender, education level, and factors such as smoking, alcohol consumption, physical activity, and overweight. Our findings can provide an essential foundation for developing health policies and identifying interventions to protect hospital employees’ health. The impact of education level on health habits and behaviors, in particular, should be considered when designing preventive health programs and awareness-raising activities, especially regarding their target audience and content. Further, more comprehensive studies are needed to validate our results and conduct more advanced analyses. Similar studies are valuable for understanding the health habits of hospital employees and developing appropriate strategies.
